# Exercise-based cardiac rehabilitation for stable angina: systematic review and meta-analysis

**DOI:** 10.1136/openhrt-2018-000989

**Published:** 2019-06-05

**Authors:** Linda Long, Lindsey Anderson, jingzhou He, Manish Gandhi, Alice Dewhirst, Charlene Bridges, Rod Taylor

**Affiliations:** 1 Institute of Health Research, University of Exeter Medical School, Exeter, UK; 2 Department of Innovation, Impact & Business, University of Exeter, Exeter, UK; 3 Department of Cardiology, Royal Devon & Exeter NHS Foundation Trust Hospital, Exeter, UK; 4 University of Exeter Medical School, Exeter, UK; 5 Farr Institute of Health Informatics Research, University College London, London, UK

**Keywords:** stable angina, cardiac rehabilitation, exercise, systematic review

## Abstract

**Objective:**

A systematic review was undertaken to assess the effects of exercise-based cardiac rehabilitation (CR) for patients with stable angina.

**Methods:**

Databases (Cochrane Central Register of Controlled Trials, MEDLINE, Embase and CINAHL) were searched up to October 2017, without language restriction. Randomised trials comparing CR programmes with no exercise control in adults with stable angina were included. Where possible, study outcomes were pooled using meta-analysis. Grading of Recommendations Assessment, Development and Evaluation was used to assess the quality of evidence. The protocol was published on the Cochrane Database of Systematic Reviews.

**Results:**

Seven studies (581 patients), with a median of 12-month follow-up, were included. The effect of exercise-based CR on all-cause mortality (risk ratio (RR) 1.01, 95 % CI: 0.18 to 5.67), acute myocardial infarction (RR 0.33, 95% CI: 0.07 to 1.63) and cardiovascular-related hospital admissions (RR 0.14, 95% CI: 0.02 to 1.1) relative to control were uncertain. We found low-quality evidence that exercise-based CR results in a moderate improvement in exercise capacity (standard mean difference 0.45, 95% CI: 0.20 to 0.70). There was limited and very low-quality evidence for the effect of exercise-based CR on health-related quality of life (HRQoL), adverse events and costs. No data were identified on cost-effectiveness or return to work.

**Conclusions:**

Exercise-based CR may improve the short-term exercise capacity of patients with stable angina pectoris. Well-designed randomised controlled trials are needed to definitely determine the impact of CR on outcomes including mortality, morbidity, HRQoL, and costs in the population of patients with stable angina receiving contemporary medical therapy.

**Key questions**

**What is already known about this subject?**

- Cardiac rehabilitation (CR) is recognised as integral to the comprehensive care of patients with coronary heart disease. CR is a process by which patients, in partnership with health professionals, are encouraged and supported to achieve and maintain optimal physical health, with exercise training at the centre of rehabilitation provision for CR. There is an absence of evidence and consequently inconsistency in guideline and policy recommendations for the use of CR programme for patients with stable angina. American College of Cardiology/American Heart Association and European Society of Cardiology guidelines recommend CR for patients with cardiac disease, including patients with stable angina. The 2017 British Association for Cardiovascular Prevention and Rehabilitation guidelines recommend CR for people with established forms of cardiovascular disease, including stable angina. However, the National Institute for Health and Care Excellence do not recommend CR in their clinical guidelines for the management of stable angina.

**What does this study add?**

- This systematic review assesses the impact of CR for people with stable angina, defined in this review as chest pain and associated symptoms precipitated by activity (eg, running, walking) with minimal or non-existent symptoms at rest. The review finds exercise-based CR may improve the short-term exercise capacity of people with stable angina pectoris, based on low-quality evidence. The review finds insufficient evidence to determine effectiveness of exercise-based CR on clinical relevant outcomes or quality of life.

**Key questions**

**How might this impact on clinical practice?**

- The impact of exercise-based CR for adults with stable angina is uncertain due to insufficient evidence, with the quality of the evidence graded as low to very low. However, it may be associated with a moderate increase in exercise capacity compared with no exercise control. Well-designed randomised controlled trials are required to definitively assess the impact of adding CR to contemporary usual care in terms of mortality, morbidity, health-related quality of life, and costs.

## Introduction

With increasing numbers of patients living longer with symptomatic coronary heart disease (CHD), the effectiveness and accessibility of health services for patients with CHD have never been more important. Cardiac rehabilitation (CR) is recognised as integral to the comprehensive care of patients with CHD.^1^ CR is a process by which patients, in partnership with health professionals, are encouraged and supported to achieve and maintain optimal physical health.^2^ While physical exercise training is at the centre of rehabilitation provision for CR, it is now accepted that programmes should be comprehensive in nature and also include education and psychological care, as well as focus on health and life-style behaviour change and psychosocial well-being.^3^

Stable angina is a form of chronic heart disease associated with ill health and increased death rates and is defined in this review as chest pain and associated symptoms precipitated by activity (eg, running, walking) with minimal or non-existent symptoms at rest. It was estimated that in 2013 over 1.3 million people in the UK had angina^4^ and it was thought to affect approximately 112 million people, or 1.6% of the population worldwide.^5^ Although clinical guidelines consistently recommend referral for CR for post-myocardial infarction (MI) and patients with heart failure (HF), advice for patients with stable angina is less clear. The American College of Cardiology/American Heart Association give a Class I recommendation that medically supervised CR programmes and physician-directed, home-based programme are offered to at-risk patients with stable CHD including those with stable angina, at first diagnosis.^6^ Similarly, the European Society of Cardiology recommends that people with stable CHD, including stable angina, should undergo ‘moderate-to-vigorous intensity aerobic exercise training ≥3 times a week and for 30 min per session’.^7^ However, National Health and Care Excellence (NICE) guideline for the management of stable angina (CG126) states that there is ‘no evidence to suggest that CR is clinically or cost-effective for managing stable angina’.^8^

To inform current practice and policy, we therefore sought to undertake a systematic review and meta-analysis of randomised controlled trials (RCTs) to assess the effects of exercise-based CR versus usual care on mortality, morbidity, hospital admissions, exercise capacity, health-related quality of life (HRQoL), adverse events and return to work for adults with stable angina.

## Methods

We conducted and reported this systematic review in accordance with the Preferred Reporting Items for Systematic Reviews and Meta-Analyses statement and the Cochrane Handbook for Interventional Reviews.^9^ The protocol was published on the Cochrane Database of Systematic Reviews.^10^

### Searches

We adapted the search strategy based on the Cochrane systematic review of exercise-based CR for CHD.^11^ We searched databases using a strategy combining selected Medical Subject HeadingsMeSH terms and free-text terms relating to exercise-based rehabilitation and stable angina, with filters applied to limit to RCTs. Electronic searches of the Cochrane Central Register of Controlled Trials, MEDLINE, EMBASE and CINAHL plus others (see online supplementary document A) were performed. Databases were searched up to September 2016 with no language or other restrictions, and then updated with a further search up to October 2017. Trial registers (www.who.int/ictrp/en and clinicaltrials.gov) were also checked, in addition to reference lists of all eligible studies and other published systematic reviews.

10.1136/openhrt-2018-000989.supp1 Supplementary data

### Study selection

We included randomised trials (individual or cluster) directly comparing CR programmes with a no exercise control or usual care comparison. The study population included adults with stable or exertional angina (effort-induced chest discomfort), who were being treated with medical anti-anginal therapy and who may have had a MI, coronary artery bypass graft (CABG) or percutaneous coronary intervention (PCI). We excluded patients in the immediate period following such an event that is, within 3 months of previous MI, CABG or PCI. We also excluded patients with unstable angina (pain at rest) and those with refractory angina for whom revascularisation was planned.

Studies with one or more of the following outcome measures with ≥6 month-follow-up were included: mortality (cardiac and overall); morbidity (reinfarction, revascularisation or cardiac-related hospitalisation); exercise capacity; HRQoL, adverse events (withdrawal from the trial or exercise programme); return to work. Selection of studies involved the initial screening of titles and abstracts, followed by an assessment of the full-text reports of all potentially relevant trials. Two authors (AD and LA) independently assessed trials for inclusion and where there was a disagreement, the opinion of a third author (RST, GH or MG) was sought.

### Data extraction and risk of bias assessment

The following information was extracted: study design, participants (baselinecharacteristics), details of the intervention (including type, frequency, duration and intensity of exercise training and nature of co-interventions), length of follow-up and outcome results. We assessed study risk of bias using the Cochrane standard criteria^9^ (random sequence generation and allocation concealment, dropouts and withdrawals, outcome blinding, and selective reporting) and two further items deemed relevant to this review (balance of groups at baseline and if the study groups received comparable care (apart from the exercise component of the intervention)). These criteria, agreed on in advance by the review authors, have not been validated but have been used to assess quality in previous CR reviews^11–15^

Data extraction and risk of bias assessment were carried out independently by three authors (LL, JH and AD). Any disagreements were resolved by consensus and decisions were independently checked by a third author (RST). Where necessary, authors of included studies were contacted for further information (eg, when a study was identified as abstract only).

### Data analysis

Data were analysed in accordance with the Cochrane Handbook.^9^ For dichotomous variables, relative risks (RRs) and 95% CIs were calculated for each outcome, and for continuous variables, mean differences (MDs) and 95% CIs were calculated. Given the variety of outcome measures reported for exercise capacity, to allow us to pool findings across studies, between-group results for each study were expressed as a standard MD (SMD). Where differences between groups for each individual trial were not reported, we calculated p values for the differences.

We explored heterogeneity among the included studies qualitatively (by comparing the study characteristics) and quantitatively (using the χ^2^ test of heterogeneity and I^2^ statistic). Where appropriate, an overall estimate of treatment effect was obtained by combining the results from included studies for each outcome. We employed a random-effects model where there was formal evidence of statistical heterogeneity (ie, χ^2^ test p value<0.10 and I^2^ statistic of 50%–90%). For outcomes with lower levels of statistical heterogeneity, we applied both fixed and random effects models, reporting fixed-effects results unless there was a difference in statistical inference, where we reported the most conservative random-effects model. We sought to explore small-study bias and the potential for publication bias using funnel plot and the Egger test^16^

Analyses were undertake using Review Manager Software V.5.3 (Nordic Cochrane Group, Copenhagen, Denmark).

### Summary of findings table

Two reviewers (LL and RST) independently employed the Grading of Recommendations Assessment, Development and Evaluation (GRADE) approach^17^ to interpret result findings. We used the five GRADE considerations (study limitations, consistency of effect, imprecision, indirectness and publication bias) to assess the quality of a body of evidence from studies that contributed data to the meta-analyses and narrative summaries for the pre-specified outcomes. Any discrepancies in judgements were resolved through discussion. One reviewer (LL) used GRADEpro GDT 2015 to import data from Review Manager to create a ‘Summary of findings’ table using the following pre-specified outcomes: all-cause mortality; MI; all-cause hospital admissions; HRQoL, return to work and exercise capacity.

## Results

### Study selection

**Figure 1:**
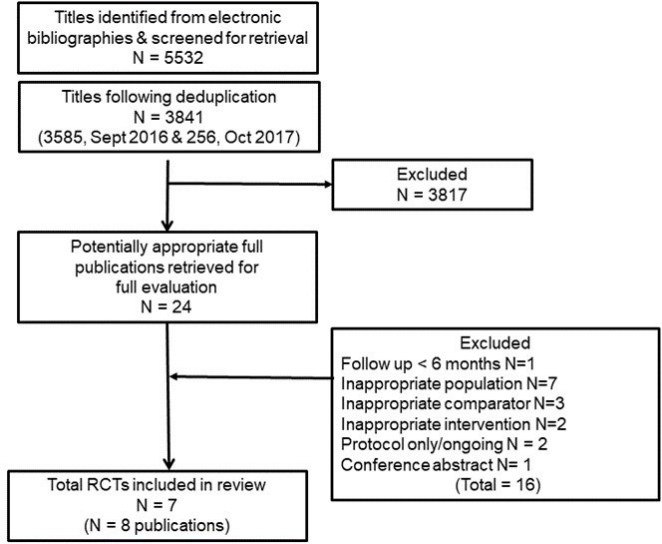
Summary of study selection process. RCT, randomised controlled trials.

Figure 1 summarises the screening process resulting in eight publications across seven RCTs included in the review.

### Characteristics of included studies

The seven RCTs included a total of 581 patients with stable angina. A summary of study characteristics is shown in tables 1 and 2.

**Table 1:** Summary of individual studies

Study	Participants (number and % men)	Intervention, comparator and setting	Exercise prescripti0m	Outcomes	Follow-up	Country/setting
Devi *et al* 2014^23^	94 stable angina pectoris; 74% men	Home-based online web-based intervention	Dose: Individualised daily exercise (most commonly walking) Length of session: Not reported Frequency: Daily Intensity: Moderate Total duration: 6 weeks	HRQoL and anxiety and depression	6 months	UK, single centre
Hambrecht *et al* 2004^21^	101 classes I to III angina pectoris; 100% men	Home-based aerobic training (bicycle ergometer)	Dose: 48×7×20 min Length of session: 20 min Frequency: Daily Intensity: Not reported Total duration: 12 months	Angina symptoms (CCS), exercise capacity, revascularisations, MI, cost- effectiveness, combined clinical endpoint (death cardiac, stroke, CABG, PCI, AMI, worsening angina with objective evidence resulting in hospitalisation)	12 months	Germany, single centre
Jiang et al 2007	167 first hospitalised with angina pectoris or MI; 71.2% men	Hospital-based patient/family education and home-based rehabilitation care	Dose: Not reported Length of session: Not reported Frequency: Not reported Intensity: Not reported Total duration: 12 weeks	None relevant to this review	6 months	China, single centre
Manchanda *et al* 2000^22^	42 chronic stable angina pectoris and coronary artery disease (CAD); 100% men	Home-based yoga lifestyle intervention programme	Dose: 48×7×90 min Length of session: 90 min Frequency: Daily Intensity: Moderate Total duration: 12 months	All-cause mortality, severity of angina, revascularisation, exercise capacity	12 months	India, single centre
Raffo *et al* 1980^18^	24 stable angina pectoris; 88% men	Hospital-based aerobic training (Canadian Air Force Programme)	Dose: 24×7×11 Length of session: 11–12 min (daily) Frequency: Daily (at home) Intensity: Training started at lowest physical capacity level, and progressed by increasing this level according to their age and sex. Total duration: 6 months	Exercise capacity	6 months	Country not reported, single centre
Schuler *et al* 1992^20^	113 stable angina pectoris; 100% men	Home-based aerobic training (bicycle ergometer)	Dose: 48×7×30 min (daily exercise) plus 48×2×60 min (weekly exercise) Length of session: 30 min minimum Frequency: Daily Intensity: 75% maximal heart rate during symptom-limited exercise Total duration: 12 months	All-cause mortality, MI, revascularisations, exercise capacity, adverse events	12 months	Germany, single centre
Todd *et al* 1991^19^	40 chronic stable angina >6 months duration	Home-based aerobic training (Canadian Air Force Programme)	Dose: 48×7×11 Length of session: 11 min Frequency: Daily Intensity: Increasing intensity with no limit on maximum exercise level Total duration: 12 months	All-cause mortality, MI, exercise capacity	12 months	UK, single centre

AMI, acute myocardial infarction; CABG, coronary artery bypass graft; CCS, Canadian Cardiovascular Society; HRQoL, health-related quality of life; MI, myocardial infarction; PCI, percutaneous coronary intervention.

**Table 2:** Summary of study characteristics

	Number of studies (%) or median (range)
Study characteristics	
Publication year	
1980–1999	3
2000–2009	3
2010 onwards	1
Study location	
Europe	5
Australasia	2
Single centre	7
Sample size	126 (24–167)
Duration of follow-up	9.4 months (6–12)
Comparator	
Usual medical care	6
PCI	1
Population characteristics	
Sex	
Males only	4
Both males and females	3
Age (years)	56.6 (50–66.2)
Intervention characteristics	
Intervention type	
Exercise-only programme	4
Comprehensive programme	3
Duration of intervention (months)	57.5 (1.5–12)
Nature of intervention	
Aerobic only	7
Aerobic and resistance	0
Dose of intervention	
Duration	57.5 months (1.5–12)
Frequency	1–7 sessions/week
Length	11–90 min/session
Intensity	70%–75% of maximal heart rate ‘Moderate’ intensity
Setting	
Centre-based only	1
Combination of centre- and home-based	3
Home-based only	2

PCI, percutaneous coronary intervention.

### Risk of bias

**Figure 2:**
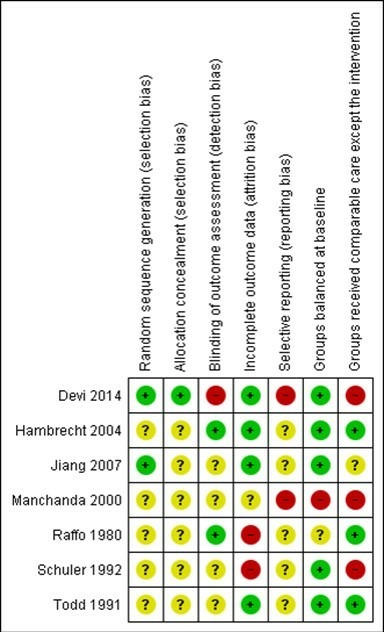
Risk of bias summary: review authors' judgements about each risk of bias item for each included study. + (green), low risk of bias; ? (yellow), unclear risk of bias; − (red), high risk of bias.

Overall risk of bias was judged to be poor, most studies being insufficiently reported to fully assess their potential risk of bias (see figure 2). The reporting of details tended to be poorer in studies published prior to 2000.^18–20^ Details of selection bias (random allocation sequence generation and concealment) and reporting bias were particularly poorly reported. Only two studies stated they took measures to blind outcome assessment.^18 21^ Loss to follow-up or dropout as well as whether groups received the same co-interventions appeared to vary considerably across studies. Where reported, losses to follow-up and drop-out were relatively high, ranging from 15% to 58% across studies. The majority of trials were judged to be of low risk of bias in terms of the risk associated with groups being unbalanced at baseline.

### Impact of CR on outcomes

#### Mortality and morbidity

Three studies^19 20 22^ (195 participants) reported a total of four all-cause deaths with a pooled RR of 1.01 (95% CI: 0.18 to 5.67, I^2^=0%, fixed effects). We are uncertain if CR has little or no effect on all-cause mortality due to very low-quality evidence (Summary of findings table 3). One study (Schuler 1992)^20^ reported cardiovascular-related mortality in two participants in the CR group and none in the control.

**Table 3:** Summary of findings table

Outcomes	Anticipated absolute effects* (95% CI)		Relative effect (95% CI)	No of participants (studies)	Quality of the evidence (GRADE)
	Risk with usual care	Risk with exercise-based CR			
All-cause mortality Follow-up: 12 months	20 per 1000	**21 per 1000** (4 to 116)	**RR 1.01** (0.18 to 5.67)	195 (3 RCTs)	⨁◯◯◯ Very low*†‡
AMI Follow-up: 12 months	39 per 1000	**13 per 1000** (3 to 64)	**RR 0.33** (0.07 to 1.63)	254 (3 RCTs)	⨁◯◯◯ Very low†‡§
Exercise capacity assessed using a variety of outcomes including VO_2_ max and duration of exercise Follow-up: range 6 months to 12 months	The mean exercise capacity in the intervention groups was 0.45 SD higher(0.2 higher to 0.7 higher)		–	267 (5 RCTs)	⨁⨁◯◯ Low¶**
Cardiovascular hospital admissions assessed with: Combined clinical endpoint (cardiac death, stroke, CABG, PCI, AMI, worsening angina with objective evidence resulting in hospitalisation) Follow-up: 12 months	Risk with usual care **140 per 1000** Risk with exercise-based CR **20 per 1000** (2–154)		**RR 0.14** (0.02 to 1.1)	101 (1 RCT)	⨁◯◯◯ Very low†††‡‡
HRQoL assessed with: Seattle angina questionnaire and The MacNew questionnaire Follow-up: range 6 weeks to 6 months	One study showed improvement in emotional score at 6 week follow-up, and benefits in angina frequency and social HRQoL score at 6 months follow-up		Not estimable	94 (1 RCT)	⨁◯◯◯ VERY LOW§§‡‡
Return to work	No studies were found that looked at return to work.		–	–	–
Adverse events Follow-up: 12 months For example, skeletomuscular injury	Only one study looked at adverse events and reported that there were no adverse events during the exercise-based CR.		Not estimable	101 (1 RCTs)	⨁◯◯◯ Very low †††‡‡

* The risk in the intervention group (and its 95% confidence interval) is based on the assumed risk in the comparison group and the relative effect of the intervention (and its 95% CI)

*Some concerns with random sequence generation, allocation concealment, blinding of outcome assessment and selective reporting; bias likely, therefore quality of evidence downgraded by one level.

†Some concern with applicability to review question as participants in all studies were limited to middle-aged men, therefore quality of evidence downgraded by one level.

‡Imprecise due to small number of participants (less than 300) and CIs including potential for important harm or benefit as 95% CI crosses RR of 0.75 and 1.25, therefore quality of evidence downgraded by two levels.

§Some concern with random sequence generation, allocation concealment, blinding of outcome assessment, high loss to follow-up, selective reporting and unbalanced groups at baseline; serious bias likely, therefore quality of evidence downgraded by two levels.

¶Some concern with random sequence generation, allocation concealment, blinding of outcome assessment, selective reporting and unbalanced groups at baseline; bias likely, therefore quality of evidence downgraded by one level.

**Imprecise due to small number of participants (less than 300), therefore quality of evidence downgraded by one level.

††Some concerns with random sequence generation, allocation concealment and selective reporting; bias likely, therefore quality of evidence downgraded by one level.

‡‡Imprecise due to very small number of participants therefore quality of evidence downgraded by two levels.

§§Some concerns with blinding of outcome assessment, selective reporting and groups not receiving comparable care; bias likely, therefore quality of evidence downgraded by one level.

AMI, acute myocardial infarction; CABG, coronary artery bypass graft; CR, cardiac rehabilitation; GRADE, Grading of Recommendations Assessment, Development and Evaluation; HRQoL, health-related quality of life; PCI, percutaneous coronary intervention; RCT, randomised controlled trial.

#### Morbidity

Three studies^19–21^ (254 participants) reported on the incidence of MI with a total of six events. There was a pooled RR for risk of MI of 0.33 (95% CI: 0.07 to 1.63, I^2^=0%, fixed effects). We are uncertain if CR has little or no effect on the incidence of MI due to very low-quality evidence (Summary of findings table 3).

Three studies^20–22^ (256 participants) reported on the incidence of revascularisations with a total of 28 events. In total, six revascularisations were reported among the CR groups in the three studies, and 22 in the control groups, with a pooled RR for risk of revascularisations of 0.27 (95% CI: 0.11 to 0.64, I^2^=0%, fixed effects). We are uncertain if CR has little or no effect on the incidence of revascularisations due to very low-quality evidence (Summary of findings table 3).

One study^21^ (101 participants) reported that one CR participant and seven control participants experienced cardiovascular-related hospital admissions (RR 0.14, 95% CI: 0.02 to 1.10). We are uncertain if CR has little or no effect on the incidence of cardiovascular-related hospital admissions due to very low-quality evidence (Summary of findings table 3). None of the included studies reported all-cause hospital admissions.

#### Exercise capacity

**Figure 3:**
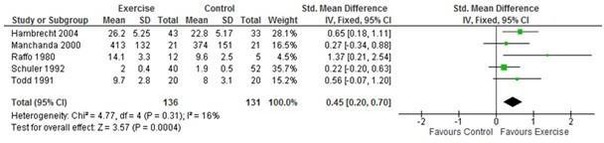
Exercise capacity with exercise-based CR versus no exercise for stable angina. CR, cardiac rehabilitation.

Five studies^18–22^ (267 participants) reported exercise capacity with a range of validated measures (peak oxygen uptake and exercise duration) (figure 3). CR may result in a moderate improvement in exercise capacity with CR compared with control in the short term (6–12 months follow-up) (SMD 0.45, 95% CI: 0.20 to 0.70; I^2^=16%, fixed effects) (figure 3) based on low-quality evidence (Summary of findings table 3). Studies varied in degree of losses to follow-up which ranged from 0%^20^ to 29%^21^ in the intervention arm.

#### HRQoL

One study^23^ (94 participants) reported HRQoL using validated disease-specific instruments (Seattle angina questionnaire and MacNew questionnaire). Compared with control, improvements with CR at the 6-week follow-up were seen in emotional score (p=0.04) and angina frequency (p=0.002). Benefits in favour of CR in angina frequency (P=0.02) and social HRQoL score (p=0.02) were also observed at the 6-month follow-up. We are uncertain of the effect of CR on HRQoL due to very low-quality evidence (Summary of findings table 3).

#### Severity of angina

One study^22^ (42 participants) reported a reduction in mean New York Heart Association (NYHA) score from baseline to 1-year follow-up (2.6–1.4, p<0.0001) with CR and an increase in mean NYHA in control (2.3–2.9, p≤0.004). Another study^21^ (101 participants) reported an improvement in angina severity assessed by mean Canadian Cardiovascular Society score in both CR (1.5–0.4, p<0.001) and control (1.7–0.7, p<0.001). We assessed the evidence as very low‐quality using GRADE because of concerns about risk of bias (random sequence generation, allocation concealment, blinding of outcome assessment, selective reporting, high losses to follow‐up and unbalanced groups at baseline), concerns about applicability to review question (participants in all studies were limited to middle‐aged men) and concerns about imprecision (small number of participants). Data could not be pooled in a meta‐analysis because of scales used to report outcome measures (ie, NYHA is categorical while CCS is continuous).

#### Adverse events

Adverse events were only reported in one study^21^ (101 participants). The authors reported ‘no adverse events’ during the exercise training programme in the CR group (summary of findings in table 3).

#### Healthcare costs

One study^21^ (101 participants) reported a difference in mean participant healthcare costs in favour of CR (CR: US$3708 vs control: US$6086, p<0.0001). These costs included hospitalisations, repeat vascularisations, any other cardiovascular events plus the costs of the provision of the CR exercise training programme. We assessed this evidence as very low‐quality using GRADE because of concerns about risk of bias (random sequence generation, allocation concealment, high losses to follow‐up and selective reporting), concerns about applicability to review question (participants in all studies were limited to middle‐aged men) and concerns about imprecision (small number of participants). We are uncertain of the effect of CR on healthcare costs due to very low-quality evidence.

#### Return to work

None of the included studies reported on return to work (summary of findings in table 3)

### Small study bias

There were insufficient (<10 RCTs) studies to assess small study bias using Funnel plot or Egger test.

## Discussion

This systematic review identified seven RCTs in 581 patients with a confirmed diagnosis of stable angina that compared exercise-based CR with a no exercise control. Meta-analysis and GRADE analysis showed there may be an improvement in exercise capacity following CR compared with control in the short term (up to 12 months follow-up, low-quality evidence). However, there was insufficient evidence to be able to definitely assess impact of CR on mortality, morbidity, or HRQoL.

The limited evidence base for CR for stable angina identified by this review is in contrast to that reported for post-MI, following coronary revascularisation, and in patients with HF. A Cochrane meta-analysis of exercise-based CR for CHD identified 63 RCTs in 14 486 patients, the majority following MI or coronary revascularisation. Compared with no-exercise control, the authors reported that CR reduced the risk of cardiovascular mortality (risk ratio (RR) 0.74, 95% CI 0.64 to 0.86 and hospital admission (RR 0.82, 95% CI 0.70 to 0.96) and improved HRQoL.^11^ A systematic review and meta-analysis comparing the effects of home-based and supervised centre-based CR found similar benefits in terms of clinical and HRQoL outcomes at equivalent cost for patients with HF and following MI and revascularisation.^24^ The precise mechanisms by which CR may improve mortality in people with CHD has not been fully elucidated. Exercise training has been shown to have direct benefits on the heart and coronary vasculature, including autonomic tone, endothelial function, myocardial oxygen demand, coagulation and clotting factors, inflammatory markers, and the development of coronary collateral vessels.^25–27^ However, it has been suggested that approximately half of the 28% reduction in cardiac mortality in people with CHD may also be mediated via the indirect effects of exercise through improvements in the risk factors for atherosclerotic disease (ie, smoking, blood pressure and total cholesterol).^28^

A recent review that focused on contemporary exercise-based CR found no improvement in all-cause mortality and potential benefit on hospital admissions.^29^

A recent Cochrane review from 2019^30^ of exercise-based CR identified 44 RCTs that included 5783 people with HF. The findings show important benefits of exercise-based rehabilitation that include a probable reduction in the risk of overall hospital admissions in the short term (RR: 0.7, 95% CI 0.6 to 0.83), as well as the potential for reduction in HF admissions (RR: 0.59 95% CI 0.42 to 0.84) compared with usual care control. The effect of exercise-based rehabilitation on HRQoL is uncertain due to very low-quality evidence. Exercise-based rehabilitation may make little or no difference in all-cause mortality in trials with follow-up less than 12 months.

### Strengths and limitations

We believe this to be the first systematic review and meta-analysis to specifically assess the impact of CR in patients with stable angina. Strengths of this review include extensive literature searches, consideration of RCTs, application of Cochrane review methodology and consideration of a wide range of outcomes. However, a major limitation was the small number of RCTs and patients included in the studies. It was therefore not possible to assess potential small study effects and publication bias. Trials generally recruited primarily younger middle-aged men,^19–22^ limiting external generalisability. A number of the trials were in patient populations with poorly defined baseline characteristics in terms of their angiographic coronary disease and left ventricular ejection fraction and were not receiving contemporary medical therapy (ie, antiplatelet, antianginal and revascularisation therapies).

### Implications for clinical practice and future research

It is estimated that stable angina is prevalent in over 1.3 million people in the UK,^4^ and in approximately 112 million people, or 1.6% of the population worldwide.^5^ The findings of this review support NICE clinical guidance that there is currently insufficient evidence to conclude whether CR is clinically effective or cost-effective for stable angina.

Adequately powered, high-quality, multi-centre randomised trials of exercise-based CR in patients with stable angina receiving contemporary medical care are required. Such trials should seek to compare CR to contemporary usual care, assessing outcomes that include symptom burden with validated angina questionnaires and HRQoL measures, report clinical events including hospital admissions, all-cause mortality, and, costs and cost-effectiveness.

## Conclusions

The results of this systematic review and meta-analysis show that exercise-based CR may improve short-term exercise capacity in patients with stable angina pectoris. There is insufficient evidence to draw conclusions for any other outcome. Given the limited body of available evidence, well-designed RCTs in a contemporary patient population are required to definitely assess the impact of adding CR to usual care in terms of mortality, morbidity, HRQoL and costs.
