# Supplementary material for: Exercise-based cardiac rehabilitation for stable angina: systematic review and meta-analysis
Source: Open Heart. 2019 Jun 5;6(1):e000989. doi: 10.1136/openhrt-2018-000989 (PMC6560669; doi:10.1136/openhrt-2018-000989)
Supplement: Supplementary data [file openhrt-2018-000989supp001.docx]

**Supplementary Document A**

**Search strategy**

CENTRAL and DARE

#1 MeSH descriptor: [Angina Pectoris] this term only

#2 MeSH descriptor: [Angina, Unstable] this term only

#3 angina*

#4 stenocardia*

#5 angor pectoris

#6 #1 or #2 or #3 or #4 or #5

#7 MeSH descriptor: [Exercise Therapy] explode all trees

#8 MeSH descriptor: [Sports] this term only

#9 MeSH descriptor: [Physical Exertion] this term only

#10 rehabilitat*

#11 (physical* near/5 (fit* or train* or therap* or activit*))

#12 MeSH descriptor: [Exercise] explode all trees

#13 (train* near/5 (strength* or aerobic* or exercise*))

#14 ((exercise* or fitness) near/3 (treatment or intervent* or program*))

#15 MeSH descriptor: [Rehabilitation] explode all trees

#16 kinesiotherap*

#17 MeSH descriptor: [Physical Education and Training] this term only

#18 MeSH descriptor: [Patient Education as Topic] this term only

#19 (patient* near/5 educat*)

#20 ((lifestyle or life‐style) near/5 (interven* or program* or treatment*))

#21 MeSH descriptor: [Self Care] this term only

#22 (self near/5 (manag* or care or motivate*))

#23 MeSH descriptor: [Psychotherapy] explode all trees

#24 psychotherap*

#25 (psycholog* near/5 intervent*)

#26 MeSH descriptor: [Counseling] this term only

#27 (counselling or counseling)

#28 ((behavior* or behaviour*) near/5 (modify or modificat* or therap* or change))

#29 (psycho‐educat* or psychoeducat*)

#30 (motivat* near/5 (intervention or interv*))

#31 MeSH descriptor: [Health Education] this term only

#32 (health near/5 educat*)

#33 (psychosocial or psycho‐social)

#34 (cognitive near/2 behav*)

#35 #7 or #8 or #9 or #10 or #11 or #12 or #13 or #14 or #15 or #16 or #17 or #18 or #19 or #20 or #21 or #22 or #23 or #24 or #25 or #26 or #27 or #28 or #29 or #30 or #31 or #32 or #33 or #34

#36 #6 and #35

MEDLINE

1 angina pectoris/ or angina, stable/

2 angina.tw.

3 stenocardia*.tw.

4 angor pectoris.tw.

5 1 or 2 or 3 or 4

6 exp Exercise Therapy/

7 Sports/

8 Physical Exertion/

9 rehabilitat*.tw.

10 (physical* adj5 (fit* or train* or therap* or activit*)).tw.

11 exp Exercise/

12 (train* adj5 (strength* or aerobic* or exercise*)).tw.

13 ((exercise* or fitness) adj3 (treatment or intervent* or program*)).tw.

14 exp Rehabilitation/

15 kinesiotherap*.tw.

16 "Physical Education and Training"/

17 Patient Education as Topic/

18 (patient* adj5 educat*).tw.

19 ((lifestyle or life‐style) adj5 (interven* or program* or treatment*)).tw.

20 Self Care/

21 (self adj5 (manag* or care or motivate*)).tw.

22 exp Psychotherapy/

23 psychotherap*.tw.

24 (psycholog* adj5 intervent*).tw.

25 Counseling/

26 (counselling or counseling).tw.

27 ((behavior* or behaviour*) adj5 (modify or modificat* or therap* or change)).tw.

28 (psycho‐educat* or psychoeducat*).tw.

29 (motivat* adj5 (intervention or interv*)).tw.

30 Health Education/

31 (health adj5 educat*).tw.

32 (psychosocial or psycho‐social).tw.

33 (cognitive adj2 behav*).tw.

34 6 or 7 or 8 or 9 or 10 or 11 or 12 or 13 or 14 or 15 or 16 or 17 or 18 or 19 or 20 or 21 or 22 or 23 or 24 or 25 or 26 or 27 or 28 or 29 or 30 or 31 or 32 or 33

35 randomized controlled trial.pt.

36 controlled clinical trial.pt.

37 randomized.ab.

38 placebo.ab.

39 drug therapy.fs.

40 randomly.ab.

41 trial.ab.

42 groups.ab.

43 35 or 36 or 37 or 38 or 39 or 40 or 41 or 42

44 exp animals/ not humans.sh.

45 43 not 44

46 5 and 34 and 45

Embase

1. angina pectoris/ or stable angina pectoris/

2. angina.tw.

3. stenocardia*.tw.

4. angor pectoris.tw.

5. 1 or 2 or 3 or 4

6. exp kinesiotherapy/

7. sport/

8. exp exercise/

9. rehabilitat*.tw.

10. (physical* adj5 (fit* or train* or therap* or activit*)).tw.

11. (train* adj5 (strength* or aerobic* or exercise*)).tw.

12. ((exercise* or fitness) adj3 (treatment or intervent* or program*)).tw.

13. kinesiotherap*.tw.

14. exp rehabilitation/

15. physical education/

16. patient education/

17. (patient* adj5 educat*).tw.

18. ((lifestyle or life‐style) adj5 (interven* or program* or treatment*)).tw.

19. self care/

20. (self adj5 (manag* or care or motivate*)).tw.

21. exp psychotherapy/

22. psychotherap*.tw.

23. (psycholog* adj5 intervent*).tw.

24. counseling/

25. (counselling or counseling).tw.

26. ((behavior* or behaviour*) adj5 (modify or modificat* or therap* or change)).tw.

27. (psycho‐educat* or psychoeducat*).tw.

28. (motivat* adj5 (intervention or interv*)).tw.

29. health education/

30. (health adj5 educat*).tw.

31. (psychosocial or psycho‐social).tw.

32. (cognitive adj2 behav*).tw.

33. 6 or 7 or 8 or 9 or 10 or 11 or 12 or 13 or 14 or 15 or 16 or 17 or 18 or 19 or 20 or 21 or 22 or 23 or 24 or 25 or 26 or 27 or 28 or 29 or 30 or 31 or 32

34. random$.tw.

35. factorial$.tw.

36. crossover$.tw.

37. cross over$.tw.

38. cross‐over$.tw.

39. placebo$.tw.

40. (doubl$ adj blind$).tw.

41. (singl$ adj blind$).tw.

42. assign$.tw.

43. allocat$.tw.

44. volunteer$.tw.

45. crossover procedure/

46. double blind procedure/

47. randomized controlled trial/

48. single blind procedure/

49. 34 or 35 or 36 or 37 or 38 or 39 or 40 or 41 or 42 or 43 or 44 or 45 or 46 or 47 or 48

50. (animal/ or nonhuman/) not human/

51. 49 not 50

52. 5 and 33 and 51

CINAHL

S46 S5 AND S33 AND S45

S45 S34 OR S35 OR S36 OR S37 OR S38 OR S39 OR S40 OR S41 OR S42 OR S43 OR S44

S44 TX allocat* random*

S43 (MH "Quantitative Studies")

S42 (MH "Placebos")

S41 TX placebo*

S40 TX random* allocat*

S39 (MH "Random Assignment")

S38 TX randomi* control* trial*

S37 TX ( (singl* n1 blind*) or (singl* n1 mask*) ) or TX ( (doubl* n1 blind*) or (doubl* n1 mask*) ) or TX ( (tripl* n1 blind*) or (tripl* n1 mask*) ) or TX ( (trebl* n1 blind*) or (trebl* n1 mask*) )

S36 TX clinic* n1 trial*

S35 PT Clinical trial

S34 (MH "Clinical Trials+")

S33 S6 OR S7 OR S8 OR S9 OR S10 OR S11 OR S12 OR S13 OR S14 OR S15 OR S16 OR S17 OR S18 OR S19 OR S20 OR S21 OR S22 OR S23 OR S24 OR S25 OR S26 OR S27 OR S28 OR S29 OR S30 OR S31 OR S32

S32 TX (cognitive n2 behav*)

S31 TX (psychosocial or psycho‐social)

S30 TX (health n5 educat*)

S29 (MH "Health Education")

S28 TX (motivat* n5 (intervention or interv*))

S27 TX (psycho‐educat* or psychoeducat*)

S26 TX ((behavior* or behaviour*) n5 (modify or modificat* or therap* or change))

S25 TX (counselling or counseling)

S24 (MH "Counseling")

S23 TX (psycholog* n5 intervent*)

S22 TX psychotherap*

S21 (MH "Psychotherapy+")

S20 TX (self n5 (manag* or care or motivate*))

S19 (MH "Self Care")

S18 TX ((lifestyle or life‐style) n5 (interven* or program* or treatment*))

S17 TX (patient* n5 educat*)

S16 (MH "Patient Education")

S15 (MH "Physical Education and Training")

S14 TX kinesiotherap*

S13 TX rehabilitat*

S12 (MH "Rehabilitation+")

S11 TX ((exercise* or fitness) n3 (treatment or intervent* or program*))

S10 TX (train* n5 (strength* or aerobic* or exercise*))

S9 TX (physical* n5 (fit* or train* or therap* or activit*))

S8 (MH "Exercise+")

S7 (MH "Sports")

S6 (MH "Therapeutic Exercise+")

S5 S1 OR S2 OR S3 OR S4

S4 TX angor pectoris

S3 TX stenocardia*

S2 TX angina

S1 (MH "Angina Pectoris") OR (MH "Angina, Unstable")

CPCI‐S

#47 AND #46 AND #4

# 47 TS=(random* or blind* or allocat* or assign* or trial* or placebo* or crossover* or cross‐over*)

# 46 #45 OR #44 OR #43 OR #42 OR #41 OR #40 OR #39 OR #38 OR #37 OR #36 OR #35 OR #34 OR #33 OR #32 OR #31 OR #30 OR #29 OR #28 OR #27 OR #26 OR #25 OR #24 OR #23 OR #22 OR #21 OR #20 OR #19 OR #18 OR #17 OR #16 OR #15 OR #14 OR #13 OR #12 OR #11 OR #10 OR #9 OR #8 OR #7 OR #6 OR #5

# 45 TS=(cognitive NEAR/2 behav*)

# 44 TS=(psychosocial or psycho‐social)

# 43 TS=(health NEAR/5 educat*)

# 42 TS=(motivat* NEAR/5 interv*)

# 41 TS=(psycho‐educat* or psychoeducat*)

# 40 TS=(behaviour* NEAR/5 change)

# 39 TS=(behaviour* NEAR/5 therap*)

# 38 TS=(behaviour* NEAR/5 modificat*)

# 37 TS=(behaviour* NEAR/5 modify)

# 36 TS=(behavior* NEAR/5 change)

# 35 TS=(behavior* NEAR/5 therap*)

# 34 TS=(behavior* NEAR/5 modificat*)

# 33 TS=(behavior* NEAR/5 modify)

# 32 TS=(counselling or counseling)

# 31 TS=(psycholog* NEAR/5 intervent*)

# 30 TS=psychotherap*

# 29 TS=(self NEAR/5 motivate*)

# 28 TS=(self NEAR/5 care)

# 27 TS=(self NEAR/5 manag*)

# 26 TS=(life‐style NEAR/5 treatment*)

# 25 TS=(life‐style NEAR/5 program*)

# 24 TS=(life‐style NEAR/5 interven*)

# 23 TS=(lifestyle NEAR/5 treatment*)

# 22 TS=(lifestyle NEAR/5 program*)

# 21 TS=(lifestyle NEAR/5 interven*)

# 20 TS=(patient* NEAR/5 educat*)

# 19 TS=kinesiotherap*

# 18 TS=(fitness NEAR/3 program*)

# 17 TS=(fitness NEAR/3 intervent*)

# 16 TS=(fitness NEAR/3 treatment)

# 15 TS=(exercise* NEAR/3 program*)

# 14 TS=(exercise* NEAR/3 intervent*)

# 13 TS=(exercise* NEAR/3 treatment)

# 12 TS=(train* NEAR/5 exercise*)

# 11 TS=(train* NEAR/5 aerobic*)

# 10 TS=(train* NEAR/5 strength*)

# 9 TS=(physical* NEAR/5 activit*)

# 8 TS=(physical* NEAR/5 therap*)

# 7 TS=(physical* NEAR/5 train* )

# 6 TS=(physical* NEAR/5 fit*)

# 5 TS=rehabilitat*

# 4 #3 OR #2 OR #1

# 3 TS=angor pectoris

# 2 TS=stenocardia*

# 1 TS=angina

WHO's ICTRP

"Cardiac rehabilitation"

rehabilitation AND "angina"

exercise AND "angina"

Clinicaltrials.gov

"Cardiac rehabilitation"

rehabilitation AND angina

exercise AND angina
